# Nuclear envelope proteins in cancer: revisiting the significance of LEM-domain proteins

**DOI:** 10.3389/fcell.2026.1789623

**Published:** 2026-04-22

**Authors:** Amie Jobe, Sameer Mirza, Ranjit Vijayan

**Affiliations:** 1 Department of Biology, College of Science, United Arab Emirates University, Al Ain, United Arab Emirates; 2 Department of Chemistry, College of Science, United Arab Emirates University, Al Ain, United Arab Emirates; 3 Zayed Center for Health Sciences, United Arab Emirates University, Al Ain, United Arab Emirates

**Keywords:** biomarker, cancer, LEM-domain proteins, nuclear envelope proteins, nucleus

## Abstract

Nuclear envelope dysfunction is increasingly recognized as a driver of cancer-associated alterations in chromatin organization, genome stability, and mechanotransduction. Among inner nuclear membrane components are the LEM-domain (LEM-D) proteins LAP2/TMPO, emerin (EMD), LEMD1, LEMD2, MAN1/LEMD3, ANKLE1, and ANKLE2. Accumulating evidence links dysregulation of these proteins to hallmark cancer processes, including cell-cycle control, epithelial–mesenchymal transition, genome instability, and therapeutic resistance. This review synthesizes recent mechanistic and translational findings on LEM-D proteins in cancer, highlighting isoform-specific functions, context-dependent oncogenic versus tumor-suppressive roles, and convergence on key pathways such as Wnt/β-catenin, PI3K/AKT, MAPK, and TGF-β signaling. Concrete evidence for prognostic value varies across the LEM-D proteins. While much of the current evidence derives from transcript-level and preclinical studies, emerging data suggest that LEM-D proteins contribute to nuclear stress adaptation and may represent context-dependent therapeutic vulnerabilities. We discuss their prognostic and predictive potential, critically evaluate limitations in current datasets, and present a unifying framework linking LEM-D dysfunction to genome instability, altered signalling, and therapy resistance. Thus, despite growing evidence of therapeutic potential, these proteins are better positioned as biomarkers to guide current therapies.

## 1 Introduction

**FIGURE 1:**
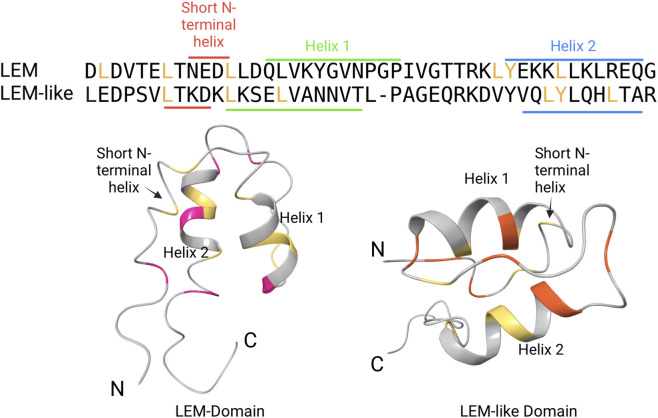
Conserved residues within the LEM (PDB ID 1H9F) and LEM-like (PDB ID 1H9E) domains of LAP2α. The sequence alignment (top) shows the conserved hydrophobic residues in yellow and the structural boundaries of the three helices. The three dimensional structure (bottom) of the two domains are shown side by side in the same camera view, illustrating their conserved three-helix architecture. Conserved core regions are highlighted in yellow, while domain-specific regions are shown in pink for the LEM and orange for the LEM-like domain.

The eukaryotic nuclear envelope (NE) consists of three interconnected major components with varying morphology—the nuclear lamina, the inner and outer nuclear membranes, and the nuclear pore complexes. The inner nuclear membrane is closely associated with the nuclear lamina, while the outer nuclear membrane is continuous with the rough and smooth endoplasmic reticulum (Lin et al., 2000). The LEM domain (LEM-D) proteins termed from the initial members—LAP2, Emerin (EMD), MAN1 — are a family of inner nuclear membrane proteins sharing a common ∼45-residue LEM-domain, a structural motif mediating association with the DNA associated protein Barrier-to-Autointegration Factor (BAF). In mammals, these proteins are encoded by 7 genes, namely, *LEMD1*, LEMD2, *LAP2*, *EMD*, *MAN1*, *ANKLE1*, and *ANKLE2*. Despite sharing only 18% similarity, both domains contain similar 3D structures, consisting of two large parallel helices and a three-residue N-terminal helix joined by conserved hydrophobic amino acids. Figure 1 shows the three-dimensional (3D) structure of the LEM-domain (PDB ID 1H9F) and the LEM-like domain of LAP2 (PDB ID 1H9E) (Laguri et al., 2001). The shared hydrophobic core maintains the same three-helix fold in both structures. In LEM, the domain-specific residues contribute to a relatively more charged solvent-exposed surface while and the LEM-like domain-specific residues define the distinct hydrophobic core organization of the LEM-like domain.

**FIGURE 2:**
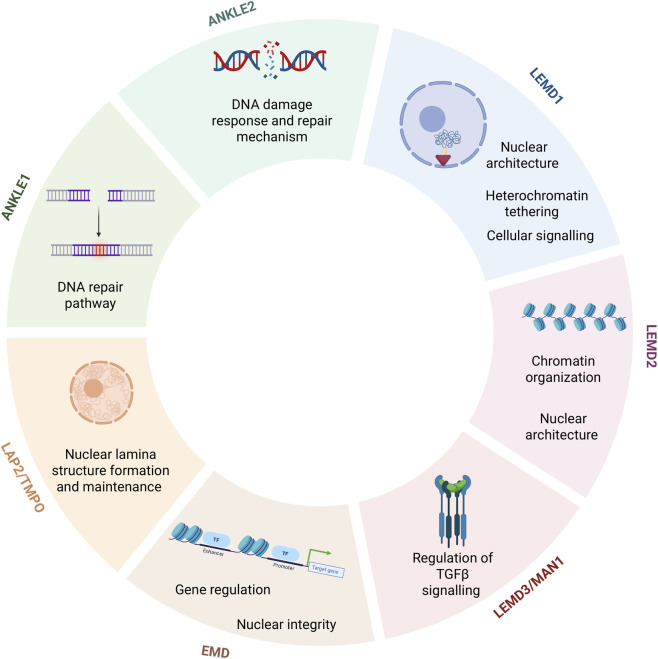
Biological functions of LEM-D proteins in normal physiology. Created with BioRender.com.

The LEM-D proteins are essential parts of the inner membrane and have one or two transmembrane domains; however, ANKLE1 lacks a transmembrane domain and shuttles between the nucleoplasm and cytoplasm, while LAP2α and γ isoforms localize to the nucleoplasm (Brachner and Foisner, 2011). Most of the LEM-D proteins connect the membrane to the lamina scaffold by binding lamins within the lamina. These proteins recruit and modulate signaling molecules (Wilson and Berk, 2010), including SMADS that are implicated in the signaling pathways of bone morphogenetic protein (BMP) and transforming growth factor beta (TGFß) (Lin et al., 2005; Pan et al., 2005), Histone deacetylase 3 (HDAC3) (Demmerle et al., 2012), the Lmo7 transcription factor (Dedeic et al., 2011), the Germ Cell Less (GCL) transcriptional repressor (Holaska et al., 2003; Nili et al., 2001), and ß-catenin, a transcriptional co-activator of the Wnt signaling pathway (Markiewicz et al., 2006). Figure 2 illustrates the main biological functions of the LEM-D proteins.

**FIGURE 3:**
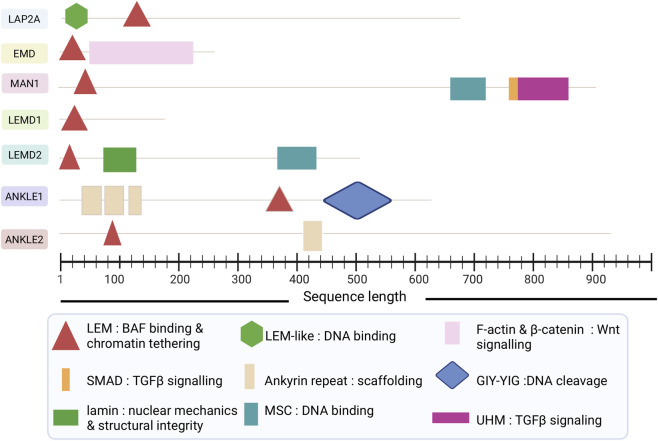
The structural organization of the LEM-D proteins highlighting the conserved LEM motif and additional functional modules. Created with BioRender.com.

Besides the conserved LEM-D which is typically located at the N-terminal end across the seven LEM-D proteins, most of the LEM-D subtypes have additional domains mostly in the C-terminal end (Figure 3). For instance, LAP2 harbors a LEM-like domain that facilitates direct DNA binding (Cai et al., 2001) while LEM2 and MAN1 carry carboxyl-terminal winged-helix ‘MSC’ (MAN1/Src1p/C-terminal) motif (Caputo et al., 2006). MAN1 has a predicted RRM-like protein interaction domain termed UHM (U2AF homology motif) (Caputo et al., 2006; Kondé et al., 2010). ANKLE1 and ANKLE2 carry ankyrin repeats which mediate protein-protein interactions (Li et al., 2006) with various structural and regulatory proteins (Demmerle et al., 2012). Other domains include the GIY-YIG nuclease domains in ANKLE1, the SMAD-binding motif in MAN1, lamin interaction motifs in LEMD2, and the β-catenin regulatory interfaces in EMD (Figure 3). These additional motifs underlie the functional diversity of LEM-D proteins in cancer, enabling integration of nuclear structure with transcriptional regulation, cytoskeletal coupling, and stress-response signaling.

## 2 Nuclear envelope mechanics, instability, and cancer progression

In cancer, NE defects are associated with chromatin dysfunction and malignant phenotype (Isermann and Lammerding, 2017; Var et al., 2012). Thus, nuclear deformation and chromatin alterations have been used for cancer diagnosis and staging for decades (Zink et al., 2004). Specifically, nuclear morphology, size, texture, and protein composition are frequently altered in malignant cells. Unlike the typically round nucleus with a smooth shape seen in normal cells, the nucleus in cancer cells may feature grooves, folds, or indentations; the chromatin may clump together or disperse; and the nucleolus may increase in size (Zink et al., 2004; Denais and Lammerding, 2014). Cells lacking particular NE proteins or expressing mutant NE proteins have similar nuclear morphological deformations, pointing to a potential link between dysregulated NE proteins and cancer pathogenesis (Zink et al., 2004; Muchir et al., 2004; Vigouroux et al., 2001). Additionally, mutations in nuclear lamina proteins disrupt nucleo-cytoskeletal connections, compromising the mechanical signaling required for migration (Friedl et al., 2011). During migration through dense extracellular matrices, cancer cells are frequently subjected to transient NE rupture (Denais et al., 2016). Such rupture events permit cytoplasmic–nuclear mixing, trigger DNA damage accumulation, and exacerbate replication stress, ultimately contributing to genomic instability. Additionally, chromosomal instability arising from nuclear defects has been shown to promote metastatic progression through cytosolic DNA sensing pathways (Bakhoum et al., 2018). Recent studies further demonstrate that micronuclei formed following NE rupture are highly prone to chromosomal rearrangements, including chromothripsis, thereby accelerating tumor evolution (Umbreit et al., 2020).

NE rupture is followed by repair processes mediated in part by the ESCRT-III machinery (Raab et al., 2016). The compromised function of this machinery can result in persistent chromatin exposure, micronuclei formation, and increased mutational load. Repeated rupture–repair cycles therefore provide a mechanistic link between nuclear mechanics and tumor evolution. Recent advances in nuclear mechanobiology further establish the nucleus as a mechanosensitive organelle that integrates cytoskeletal forces with chromatin organization (Stephens et al., 2019). More recently, emerging evidence further positions the NE as an active driver of tumor progression, showing that alterations in NE composition reshape nuclear mechanics and promote malignant transformation rather than serving solely as a diagnostic marker (Paganelli et al., 2025).

Beyond structural disruption, NE instability also bears signaling consequences. Chromatin exposure to the cytoplasm can trigger innate immune sensors such as cGAS, triggering cGAS–STING–dependent inflammatory signaling (Mackenzie et al., 2017), promoting epithelial–mesenchymal transition (EMT) and therapy resistance (Yonesaka et al., 2025). These findings indicate that NE rupture is not only a structural failure but also a driver of inflammatory signaling that may influence therapy response and immune surveillance.

**FIGURE 4:**
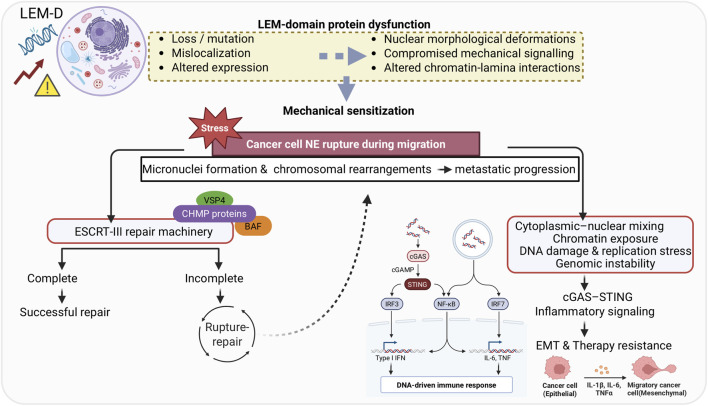
LEM-D dysfunction links nuclear mechanics to genome instability, signaling rewiring, and therapy resistance in cancer. Created with BioRender.com.

Furthermore, nuclear organization shifts can have a major effect on DNA stability and gene expression (Bickmore and van Steensel, 2013; Burke and Stewart, 2013). In particular, lamina-associated domains (LADs) represent large chromatin regions tethered to the nuclear periphery, contributing to transcriptional repression and genome organization (Van Steensel and Belmont, 2017). Within this mechanistic framework, LEM-D proteins are positioned at the interface of nuclear mechanics, chromatin topology, and genome stability. By tethering chromatin to the nuclear lamina, modulating LADs, and contributing to nuclear stiffness, LEM-D proteins may influence both susceptibility to rupture and the efficiency of NE repair mechanisms. The dysregulation of LEM-D proteins could therefore sensitize nuclei to mechanical failure, alter chromatin–lamina interactions, and promote altered signaling that supports EMT, genome instability, and therapy resistance (Figure 4). This review further delves into the implications of the LEM-D proteins in the context of cancer and highlights the therapeutic potential of such proteins for anti-cancer therapy. Table 1 summarizes LEM-D protein alterations and clinical associations in cancer.

**TABLE 1:** Summary of LEM-D protein alterations and clinical associations in cancer.

LEM-D protein	Cancer Type(s)	Alteration	Biomarker evidence	Key references
LAP2 (TMPO)	Lung, laryngeal, gastric, colorectal, pancreatic, glioblastoma, hepatic, cervical, hematologic, digestive tract, prostate, breast, CNS/embryonal tumors	Frequently upregulated (isoform-dependent)	Prognostic (lung and gastric); associative in other cancers	Agrawal et al. (2002), Huerta-Padilla et al. (2025), Kim et al. (2012), LaTulippe et al. (2002), Liu et al. (2019), Parise et al. (2006), Pomeroy et al. (2002), Somech et al. (2007), Song et al. (2025), Sun et al. (2019), Wang et al. (2024), Yokota et al. (2004), Zhang et al. (2016)
EMD	TNBC, invasive breast cancer, prostate, ovarian hepatocellular carcinoma, pancreatic (KRAS-driven)	Frequently downregulated in aggressive breast and prostate cancers (context dependent)	Prognostic (breast and prostate); associative in other cancers	Markiewicz et al. (2006), Bell and Lammerding (2016), Collins et al. (2017), Flores et al. (2025), Hansen et al. (2024), Ho et al. (2013), Liddane and Holaska (2021), Popęda et al. (2024), Przanowski et al. (2023), Reis-So et al. (2018), Watabe et al. (2021), Zhang et al. (2025)
MAN1	Glioblastoma, gastric, NSCLC, colorectal	Low-frequency mutations; context dependent downregulation	Limited clinical evidence	Lin et al. (2005), Fernandez-Rozadilla et al. (2023), Masica and Karchin (2011), Osada et al. (2003), Stewart et al. (2014), Zhou and Lu (2024)
LEMD1	Colorectal, pancreatic, TNBC, gastric, prostate, oral SCC, thyroid, NSCLC, multiple solid tumors (pan-cancer analyses)	Frequently upregulated	Prognostic (pancreatic, colorectal, NSCLC) Predictive (TNBC)	Cao et al. (2022), Li et al. (2022a); Li and Zhang (2024), Li et al. (2019), Li et al. (2023), Lin et al. (2013), Luo et al. (2021), Sasahira et al. (2016), Takeda et al. (2017), Yang et al. (2022), Zhan et al. (2022)
LEMD2	Prostate adenocarcinoma, TNBC	Upregulated	Prognostic (PRAD) Associative (TNBC)	He et al. (2022), Rose et al. (2024)
ANKLE1	Breast, ovarian, TNBC, colorectal, lung	SNP-associated cancer susceptibility (19p13.1 locus); context-dependent expression (overexpressed in TNBC; reduced in CRC)	Genetic susceptibility marker (breast and ovarian) Associative (lung and colon)	Wang et al. (2024), Przanowski et al. (2023), Antoni et al. (2010), Bakshi et al. (2021), Kang et al. (2022), Stevens et al. (2011), Tian et al. (2020)
ANKLE2	ER-positive breast, ovarian carcinoma, TNBC	Frequently upregulated	Associative (preclinical therapy resistance)	Rose et al. (2024), Al-Farsi et al. (2022), Gao et al. (2018), Tang et al. (2025)

## 3 The functional role of LEM-D proteins in cancer

### 3.1 LAP2 (TMPO)

**FIGURE 5:**
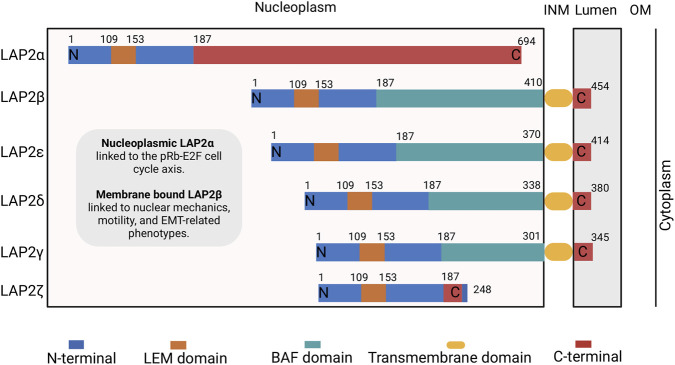
Structural organization of the six alternatively spliced LAP2 isoforms and their subcellular localization. The numbers represent the position of amino acids in human protein sequences; INM, inner nuclear membrane; OM, outer membrane. Adapted from (Dechat et al., 2000).

The mammalian LAP2 gene also referred to as thymopoietin (TMPO), encodes six splice isoforms (α, β, γ, δ, ε, ζ), all of which share a common ∼180 aa long N-terminal domain that includes an additional LEM-like motif in the N-terminus (Figure 5). The most well-understood isoforms are LAP2α and LAP2β. Nucleoplasmic LAP2α interacts with A-type lamins in the nuclear interior as part of a nucleoskeletal structure (Dechat et al., 1998; Dechat et al., 2000) while LAP2β interacts with B-type lamins at the nuclear periphery (Foisner and Gerace, 1993; Furukawa et al., 1998). The TMPO locus has also been found to be differentially expressed in the prostate (LaTulippe et al., 2002), colon (Agrawal et al., 2002) and central nervous system and embryonal tumors (Pomeroy et al., 2002; Yokota et al., 2004). The nucleoplasmic LAP2α has been reported to either repress or promote proliferation depending on the cellular context (Brachner et al., 2014; Vural et al., 2018). Several studies have demonstrated that LAP2α is mostly expressed in proliferating cells and is downregulated upon cell cycle exit and differentiation (Gotic et al., 2010; Markiewicz et al., 2002; Markiewicz et al., 2005; Naetar et al., 2008). Malignant hematologic disorders (Somech et al., 2007), digestive tract carcinomas (Kim et al., 2012), glioblastoma (Zhang et al., 2016), and various malignancies (Brachner et al., 2014) have all been associated with upregulation of LAP2α and LAP2β (Liu et al., 2019). Clinical studies have reported LAP2α overexpression as an adverse clinicopathologic and independent prognostic marker in gastric cancer (Sun et al., 2019).

Early microarray analysis of the LAP2 promoter indicated regulation by E2F1, E2F2, E2F3, p16, and retinoblastoma protein (pRb) (Müller et al., 2001; Vernell et al., 2003). Building on these finding, Parise et al. (2016) examined LAP2α expression in human tumors using tissue microarray analysis and its upregulation in significant cases of primary larynx, lung, stomach, colon and breast cancer tissues. Chromatin immunoprecipitation (ChIP) assays further demonstrated that E2F transcription factors directly regulate the LAP2 promoter, and LAP2α overexpression correlated with tumor proliferation rate in primary tumors (Parise et al., 2006). Consistently, LAP2α expression in cervical cancer was also reported to be linked to E2F-mediated regulation (Ward et al., 2011), reinforcing the connection between the LAP2α-pRb-E2F axis. Notably, as a tumor suppressor and cell cycle regulator, pRb interacts with LAP2α through its C-terminus (Markiewicz et al., 2002; Naetar et al., 2008; Dorner et al., 2006) and LAP2α has been suggested to modulate pRb location and repressor activity in cancer contexts (Markiewicz et al., 2006). Extending these observations to clinical outcome data, Liu and colleagues (Liu et al., 2019) used lung cancer clinical microarray datasets to evaluate the prognostic significance of LAP2α. They report that LAP2α is highly expressed in lung cancer tissues, with elevated levels correlating with unfavorable prognosis. The suppression of LAP2α in lung cancer cells resulted in the inhibition of cell proliferation and the induction of apoptosis. Additionally, reduced LAP2α levels were associated with decreased metastatic potential in tumor cells. Utilizing a mice tumor model, they reported that LAP2α knockdown effectively hinders tumor development *in-vivo*. These findings suggest that LAP2α may function as a context-dependent oncogenic regulator and potential prognostic marker in lung cancer (Liu et al., 2019). In a previous study, LAP2 knockdown has been shown to suppress proliferation and induce apoptosis, although isoform-specific contributions were not resolved (Zhang et al., 2016).

Interestingly, recent mechanistic work (Wang et al., 2024) has implicated LAP2α in telomere biology where LAP2α suppresses alternative lengthening of telomeres (ALT) by regulating telomeric heterochromatin via interaction with HDAC1. In ALT-positive osteosarcoma models, methotrexate curbed tumor growth, particularly in tumors with low LAP2α expression. This suggests that beyong cell-cycle regulation, LAP2α also controls telomere stability and may influence therapeutic sensitivity in ALT-dependent tumors.

The possible relevance of other LAP2 isoforms in cancer has also been explored in a few studies. LAP2ß, the largest membrane-bound LAP2 isoform, has been reported to be upregulated in various digestive tract cancers, namely, stomach, liver, bile duct and pancreatic cancer. LAP2ß knockdown in pancreatic cancer cells reduced cell motility while its ectopic expression showed the opposite effect (Kim et al., 2012). LAP2ß expression is elevated in the rapidly dividing cells of different hematological malignancies, while it is normally expressed in the slowly dividing cells associated with chronic malignant hematological disorders (Somech et al., 2007). Isoform-selective diagnostic associations have also been reported for LAP2β and γ isoforms in breast and cervical cancer samples, suggesting potential diagnostic value for specific isoforms (Marrero-Rodríguez et al., 2015). Similarly, Huerta-Padilla et al. (2025) reported LAP2 overexpression in cervical cancer, with increased expression of LAP2α,β, and γ in HPV-positive cervical (Huerta-Padilla et al., 2025) and LAP2 (not isoform-resolved) overexpression in papillary thyroid carcinoma (PTC) with predominant cytoplasmic/nuclear-membrane localization. LAP2 knockout inhibited proliferation in PTC cell lines, supporting its candidacy as a biomarker and potential therapeutic target in PTC (Song et al., 2025).

In sum, TMPO/LAP2 is often elevated across cancers, yet some evidence is transcript-based and not isoform-resolved; this distinction is critical given that LAP2α lacks a transmembrane domain and localizes to the nucleoplasm, enabling interaction with A-type lamins and the pRb–E2F cell-cycle axis, whereas membrane-bound LAP2β is anchored at the inner nuclear membrane and more closely associated with motility and EMT-related phenotypes (Figure 5). Preclinical findings indicate that upregulated LAP2 often marks proliferative drive rather than a standalone oncogenic program. However, emerging isoform-resolved studies suggest that isoform context may be particularly relevant for biomarker categorization and for defining targetable vulnerabilities in select tumor settings (Huerta-Padilla et al., 2025; Song et al., 2025; Sun et al., 2019; Wang et al., 2024; Marrero-Rodríguez et al., 2015).

### 3.2 Emerin (EMD)

Emerin is ubiquitously expressed (Holask et al., 2002; Manilal et al., 1996; Nagano et al., 1996) given its role in diverse biological functions such as transcription regulation, nuclear structure and nucleocytoskeletal mechanics, chromatin compaction, genomic organization, epigenetic modification, and cell signaling (Liddane and Holaska, 2021). A marked reduction in emerin expression has been observed in cases of triple-negative breast cancer (TNBC) (Dittmer and Misteli, 2011), and breast cancer prognosis has been reported to inversely correlate with emerin levels, with lower expression associated with reduced survival (Liddane et al., 2021). Decreased emerin expression has also been associated with enhanced migration, metastasis, and altered nuclear mechanics (Rowat et al., 2006; Lammerding et al., 2005); suggesting a role in maintaining nuclear rigidity and limiting metastatic behavior (Liddane and Holaska, 2021; Liddane et al., 2021).

Emerin depletion or mutation has also been associated with hyperactivation of Mitogen-Activated Protein Kinase (MAPK) signalling (ERK1/2), JNK, p38) MAPK (Bell and Lammerding, 2016; Collins et al., 2017; Iyer et al., 2017; Muchir et al., 2007; Muchir et al., 2008; Muchir et al., 2009), although the precise molecular mechanisms remain to be elucidated. Loss of emerin has also been associated with increased metastatic risk in breast and prostate cancer models (Reis-So et al., 2018; Hu et al., 2011; Wozniak et al., 2013). In ovarian epithelial cancer, emerin and lamin A expression correlate with subtype-specific survival patterns (Watabe et al., 2021). In breast cancer models, Hansen et al. (2024) reported that emerin deficiency promoted invasive transformation, with smaller and misshapen nuclei and an inverse correlation between emerin expression and tumor invasiveness in patient samples (Hansen et al., 2024). In contrast, in KRAS-driven pancreatic ductal adenocarcinoma, increased emerin expression was associated with reduced nuclear size and tumor progression, suggesting context-dependent regulation of nuclear dynamics in PDAC (Flores et al., 2025).

Emerin has been suggested to play an indirect role in tumorigenesis through its interactions with transcription regulators such as GCL (Holaska et al., 2003) and Lim Domain Only Protein 7 (Lmo7) (Holaska et al., 2006). GCL represses E2F-DP3 heterodimerization (delaLuna et al., 1999) and emerin downregulation may increase the expression of E2F and DP3 target genes (Holaska et al., 2003; Holaska and Wilson, 2006). Since Rb represses E2F-DP3–regulated genes required for S-phase entry, emerin has been implicated in regulating cell proliferation (Markiewicz et al., 2006). GCL also recruits GAGE proteins to the nuclear envelope in cancer cells, and is overexpressed in numerous malignancies (Gjerstorff et al., 2012; Gjerstorff and Ditzel, 2008).

Emerin binding restricts Lmo7 transcriptional activation (Holaska et al., 2006). Lmo7 overexpression has been reported across multiple cancers (Furuya et al., 2002; Kang et al., 2000; Sasaki et al., 2003). However, its precise role remains context dependent as Lmo7-deficient mice developed irregular epithelial lesions and late-onset lung adenocarcinoma at an older age, indicating that Lmo7 may function as a tumor-suppressor gene (Tanaka-Okamoto et al., 2009).

Emerin has also been been linked to major signaling pathways (Liddane et al., 2021), including Wnt, IGF, TGF-ß, and Notch (Haraguchi et al., 2004), JNK, MAPK (Collins et al., 2017). Emerin directly interacts with ß-catenin and restricts its nuclear accumulation (Markiewicz et al., 2006). Cells lacking emerin exhibit heightened expression of ß-catenin levels and transcriptional activity (Markiewicz et al., 2006; Tilgner et al., 2009). Notably, the suppression of ß-catenin correlates with reduced mRNA expression and nuclear accumulation of emerin (Tilgner et al., 2009), suggesting a reciprocal regulation of expression, localization, and function between emerin and ß-catenin. Wnt activation has been linked to EMT, stem-like cell expansion, immune evasion, and therapy resistance (Rosner et al., 2002; Spranger et al., 2015). Through regulation of nuclear actin dynamics, emerin influences MKL1/SRF signaling (Ho et al., 2013; Miralles et al., 2003), which contributes to EMT and tumor progression (Hirano and Matsuura, 2011; Pawłowski et al., 2010).

Recent studies further extend emerin’s role in cancer biology. For instance, Popęda et al. (2024) showed that emerin mislocalization to micronuclei was linked to increased invasiveness and poorer prognosis in prostate cancer, especially in metastatic tumors with higher Gleason scores (Popęda et al., 2024). Consistently, reduced EMD expression in metastatic castration-resistant prostate cancer has been linked to a very-small-nuclear phenotype in circulating tumor cells, accompanied by lineage plasticity, enhanced invasion, and resistance to androgen receptor–targeted therapies (Zhang et al., 2025).

In short, emerin downregulation is associated with worsened survival and increased migration/metastasis. Emerging evidence in prostate cancer further correlates emerin mislocalization or reduced expression to invasive phenotypes, lineage plasticity, and therapeutic resistance. Emerin contributes to nucleo-cytoskeletal coupling and nuclear mechanics and has been shown to restrain oncogenic signaling by restricting nuclear β-catenin (Wnt/β-catenin) and modulating MKL1/SRF and MAPK pathways. Clinically, evidence is strongest in breast and prostate cancers but remains heterogeneous and lineage-dependent (with potential lamin A/C confounding). Therapeutic strategies targeting Wnt/β-catenin, hyperactive MAPK activity, or plasticity programs in the context of emerin loss warrant further investigation.

### 3.3 LEMD1

LEMD1 belongs to the cancer/testis antigen (CTA) gene family (Yuki et al., 2004), which is typically expressed in normal testis and malignant tissues (Chang et al., 2019). CTAs have been associated with dysregulation in multiple cancers and have been proposed as potential tumor markers for screening, prognosis, disease progression, and therapeutic interventions (Li et al., 2017; Salmaninejad et al., 2016). Elevated CTA expression correlates with advanced disease and poorer survival outcomes (Salmaninejad et al., 2016; Mooney et al., 2016; Yang et al., 2015). LEMD1 was initially identified as overexpressed in colorectal tumors (Yuki et al., 2004), prostate cancer (Ghafouri-Fard et al., 2010), and lymphoma cells (Matsuyama et al., 2011).

Recently, Cao and colleagues (Cao et al., 2022) reported evidence supporting a potential oncogenic role for LEMD1 in pancreatic cancer. LEMD1 was upregulated and negatively associated with overall and disease-free survival rates. Functional studies showed that LEMD1 suppression reduced proliferation, migration, and invasion; whereas overexpression was associated with increased tumor aggressiveness. Gene Set Enrichment Analysis (GSEA) suggested that LEMD1 may contribute to pancreatic cancer progression via p53 repression and mTORC1 activation (Cao et al., 2022).

LEMD1 has also been implicated in prostate, oral squamous cell carcinoma, gastric, and colorectal cancers (Li et al., 2019; Sasahira et al., 2016; Yuki et al., 2004; Ghafouri-Fard et al., 2010). In thyroid cancer, LEMD1 depletion inhibited cell proliferation and migration and triggered apoptosis by suppressing EMT and Wnt/ß-catenin signaling (Xu et al., 2021). Elevated LEMD1 expression has been associated with lymph node metastasis and a dismal prognosis in oral squamous cell carcinoma (Sasahira et al., 2016; Sasahira et al., 2020). In colorectal cancer, LEMD1 upregulation correlates with poorer survival (Martinez-Romero et al., 2018), although the underlying regulatory mechanisms remain unclear.

SRY-related high-mobility-group box 4 (SOX4), a frequently overexpressed transcription factor in various malignant tumors (Hanieh et al., 2020) has been proposed as a regulator of LEMD1 expression. A recent study reported that SOX4 binds the LEMD1 promoter and that LEMD1 activation was associated with increased PI3K/AKT signaling in colon cancer (Li et al., 2022a), gastric and colorectal cancer (Li et al., 2019; Luo et al., 2021) and in various other cancer malignancies (Narayan and ankutty, 2019; Pal and Mandal, 2012; Ponnurangam et al., 2013). Recent data further reinforces PI3K/AKT pathway involvement in non-small cell lung cancer, where LEMD1 overexpression correlated with advanced stage and poorer survival and promoted malignant stemness and invasion (Li and Zhang, 2024). Additional studies indicate that LEMD1 may influence colorectal cancer stemness and motility (Takeda et al., 2017). More recently, LEMD1 was shown to promote migration in colorectal cancer through the RhoA/ROCK signaling pathway; inhibition of RhoA/ROCK attenuated LEMD1-dependent motility (Zhan et al., 2022).

In TNBC, LEMD1 was reported to be upregulated, and its silencing inhibited proliferation and migration *in-vitro* and suppressed tumor development *in-vivo* (Li et al., 2023). LEMD1 has been reported to regulate TNBC progression via ERK signaling, and its inhibition enhanced chemosensitivity. A pan-cancer analysis further demonstrated LEMD1 upregulation across multiple malignancies, with high expression associated with poorer overall survival in pancreatic adenocarcinoma, kidney renal papillary cell carcinoma, colon adenocarcinoma, and kidney renal clear cell carcinoma. Emerging evidence suggests that LEMD1 expression is regulated by the antisense lncRNA LEMD1-AS1. In OSCC, LEMD1-AS1 upregulation stabilized LEMD1 transcripts and activated PI3K/AKT signaling, enhancing metastasis (Li et al., 2022b). Conversely, reduced LEMD1-AS1 expression in epithelial ovarian cancer was associated with poorer survival, suggesting context-dependent regulation of the LEMD1 axis (Yang et al., 2022).

Overall, LEMD1 is frequently elevated across multiple cancers, and its silencing in TNBC limits tumor growth and heightens chemosensitivity, supporting a biological rationale for its potential role as a prognostic and predictive biomarker in TNBC. Functional studies suggest that LEMD1 promotes tumor progression through PI3K/AKT, ERK, and RhoA/ROCK signaling, contributing to EMT, stemness, and motility. Recent report of lncRNA-mediated regulation (e.g., LEMD1-AS1) further supports this oncogenic axis.

### 3.4 LEMD2

LEMD2 mediates chromatin-NE interactions (Brachner et al., 2005; Huber et al., 2009; Ulbert et al., 2006) and is required for NE reformation following cell division, as well as for maintaining nuclear integrity and chromatin stabilization (Vietri et al., 2020; Von Appen et al., 2020). LEMD2, together with ANKLE1 and EMD, has been reported to correlate with advanced tumor stage and survival prognosis in prostate adenocarcinoma (PRAD). Consistent with computational analysis, mRNA and protein expression levels of these genes were markedly increased in PRAD, and their expression was associated with immune cell infiltration. DNA methylation or/and copy number variations have been proposed to contribute to the upregulation of these LEM-D proteins in PRAD (He et al., 2022).

Given the frequent dysregulation of NE proteins in cancer cells, a recent study (Rose et al., 2024) evaluated the association between LEM-D proteins and TNBC. Using publicly available data, they studied the expression and prognostic implications of ANKLE2, LAP2, emerin, and LEMD2. siRNA-mediated depletion of individual LEM-D proteins was followed by proliferation and apoptosis assays. LEM-D transcripts were generally upregulated in patient samples; although protein levels were variable in TNBC cell lines and inversely associated with survival. Depletion of LEM-D proteins induced abnormal nuclear morphology, reduced proliferation, and cell death, whereas minimal effects were observed in non-cancerous cells. While these findings support an association between Ankle2, TMPO, emerin, and LEMD2 expression and TNBC, a larger cohort is needed for confirmation.

In sum, LEMD2 preserves nuclear integrity by anchoring chromatin to the NE. LEMD2 is associated with higher stage, poorer survival, and immune cell infiltration in prostate adenocarcinoma. In TNBC, transcripts levels trend upwards, whereas protein levels vary; siRNA knockdown selectively perturbs cancer nuclear morphology and proliferation, implying dependence. Therapeutic approaches targeting NE-stress or DNA damage response DDR vulnerabilities remain exploratory and warrant further investigation in LEMD2-high tumors.

### 3.5 MAN1 (LEMD3)

MAN1/LEMD3 is the longest member of the LEM family. A significant part of its role is attributed to its interaction with R-SMADs (Osada et al., 2003). The R-SMAD proteins play a crucial role as regulators of numerous signaling pathways. In addition to its role in transcriptional regulation, LEMD3 also contributes to NE organization during the cell cycle (Yam et al., 2013). It has also been reported to activate the BMAL1 promoter, a core “clock gene” that orchestrates the circadian rhythm (Lin et al., 2014).

LEMD3 counteracts TGFβ/BMP signaling (Lin et al., 2005; Osada et al., 2003) by binding R-SMADs. Since TGFβ/BMP is growth-suppressive at early stages but pro-EMT/pro-metastatic in advanced disease, LEMD3’s effect may be context-dependent, potentially modulating stage-specific signaling effects.

Although LEMD3 is not well studied as a cancer gene, recurrent mutations have been identified in specific gliomas. Assessing the impact of infrequent variants on progression is challenging. Masica and Karchin highlighted low-frequency mutated genes in The Cancer Genome Atlas (TCGA) glioblastoma (GBM) cohort. Notably, ATM, KLF6, and LEMD3 were among the mutations with low frequency in TCGA GBM tumor samples, showing complete co-mutation overlap. These mutations are strongly associated with significant altered expression of 165 additional genes, suggesting a potential cooperative role in tumorigenesis within select TCGA GBM samples (Masica and Karchin, 2011). In non-small cell lung cancer, pharmacogenetic analyses have linked LEMD3 expression and specific SNP-associated eQTL signals with overall survival in non-smokers (Stewart et al., 2014). Large-scale multi-omic analyses of colorectal cancer further identified LEMD3 among genes associated with CRC susceptibility, suggesting involvement of TGFβ-related pathways; although functional validation remains limited (Fernandez-Rozadilla et al., 2023). Provide the complete details for reference “Pamer, 2016”. https://www.frontiersin.org.cn/authors-proof-support/#QA49

LEMD3 is most notable for its implication in osteopoikilosis (OPK) and Buschke-Ollendorff syndrome (BOS). The identification of heterozygous, loss-of-function, germline mutations in the LEMD3 gene has shed light on the etiology of both OPK and BOS (Hellemans et al., 2004). OPK, a rare benign bone disorder, manifests as asymptomatic dense bone lesions that resemble bone metastasis. These mutations can arise sporadically or via autosomal dominant inheritance (Gutierrez et al., 2015). BOS is characterized by OPK along with connective-tissue nevi/juvenile elastomas, resulting in soft-tissue and skin lesions (Zhang et al., 2009). Subsequent research confirmed and expanded the spectrum of loss-of-function LEMD defects (Hellemans et al., 2006; Mumm et al., 2007). Genetic diversity has also been reported (Yadegari et al., 2010).

Although no direct link between OPK and cancer predisposition has been established, an interesting case involved a patient with an LEMD3 germline mutation and a heterozygous somatic KRAS mutation in a skin nevus. The potential facilitation of postzygotic mosaicism of mutated KRAS by germline LEMD3 haploinsufficiency remains uncertain (Hellemans et al., 2004). Recently, Correa Llano and colleagues reported a case of a patient diagnosed with a mixed germ cell tumor and OPK who achieved complete remission following chemotherapy and surgery (Correa et al., 2020). Consistent with its role as a TGFβ pathway antagonist, LEMD3 has recently been implicated in tumor microenvironment signaling. In gastric cancer, exosomal miR-21-5p was shown to target LEMD3 in endothelial cells, resulting in activation of TGFβ/Smad signaling and elevated VEGFA expression, thereby promoting angiogenesis (Zhou and Lu, 2024).

In brief, evidence for the prognostic value of MAN1/LEMD3 in cancer remains limited. Emerging studies suggest that LEMD3 may influence tumor biology through modulation of TGFβ/BMP signaling, angiogenesis, and genetic susceptibility pathways, but these associations are largely context-dependent and require further mechanistic validation. While germline loss-of-function mutations in LEMD3 underlie OPK/BOS syndromes, a definitive link to cancer predisposition has not been established.

### 3.6 ANKLE1

ANKLE1 was initially considered an endonuclease (Brachner et al., 2012). It mediates mitochondrial DNA cleavage and is involved in DNA damage response and repair mechanisms.

Given that many breast cancer-predisposition genes are involved in DNA repair pathways (e.g., BRCA1, BRCA2, Rad51, Chek2, ATM, p53), ANKLE1 has been proposed as a candidate sesceptibility gene (Brachner et al., 2014). Genomic analyses identified single nucleotide polymorphisms (SNPs) at chromosomal locus 19p13.11 siginificantly associated with breast cancer susceptibility (Antoni et al., 2010; Stevens et al., 2011; Bolton et al., 2010; Chen et al., 2011; Zhang et al., 2011). Subsequent studies reported associations between ANKLE1 variants and breast (Stevens et al., 2011) and ovarian cancer risk (Lawrenson et al., 2016). However, integrative GWAS and eQTL analyses suggests that ABHD8, rather than ANKLE1, may represent the primary causal gene at this locus (Lawrenson et al., 2016), although ANKLE1 expression may be influenced by regulatory variants affecting transcription factor binding (Liu et al., 2017). Whole-exome sequencing and genotyping studies in breast cancer cohorts from an Indian population further suggested that ANKLE1 variation may influence DNA repair efficiency. In MCF-7 cells, ANKLE1 depletion increased DNA damage and nuclear localization was observed following genotoxic stress (Bakshi et al., 2021), supporting a role in genome maintenance.

Mechanistically, elevated ANKLE1 has been reported to induce mtDNA cleavage, triggering mitophagy and a metabolic shift toward glycolysis, favoring cancer cell survival and proliferation. mtDNA degradation has also been associated with STAT1 activation and induction of EMT-related genes. In TP53-mutant contexts, ANKLE1 has been shown to cleave nuclear DNA without inducing apoptosis, potentially increasing mutational burden (Przanowski et al., 2023). These findings suggest that ANKLE1 overexpression may contribute to genomic instability and tumor progression in certain cases. Conversely, an m6A-associated variant (rs8100241) within ANKLE1 has been linked to reduced colorectal cancer (CRC) risk through increased m6A modification and elevated ANKLE1 expression (Tian et al., 2020). In CRC, ANKLE1 appears to act as a tumor suppressor by preserving genomic stability, and lower expression has been reported in tumor tissues relative to normal samples. Subsequent work has further connected ANKLE1 to m6A-dependent regulation, showing that METTL3-mediated methylation enhances ANKLE1 mRNA stability and protein expression, contributing to reduced CRC risk (Li et al., 2021). These findings underscore lineage-dependent effects, with ANKLE1 exhibiting oncogenic features in breast/TNBC models but tumor-suppressive associations in CRC. ANKLE1 has also been incorporated into prognostic gene signatures in lung squamous cell carcinoma (Jiang et al., 2022) and colon cancer (Kang et al., 2022), although functional validation remains limited.

More recently, ANKLE1 has been shown to localize to the midbody and resolve chromatin bridges during cytokinesis, thereby preventing micronuclei formation and limiting activation of the cGAS–STING pathway (Jiang et al., 2023). ANKLE1 knockout resulted in increased DNA damage, prolonged DNA damage response signaling, and replication stress, further supporting its role in genome stability maintenance.

In summary, ANKLE1 at locus 19p13.1 is associated with cancer susceptibility and DNA damage responses. Evidence suggests context-dependent roles whereby elevated expression may promote genomic instability in breast/TNBC models, while increased expression in CRC may correlate with reduced cancer risk.

### 3.7 LEM4 (ANKLE2)

**FIGURE 6:**
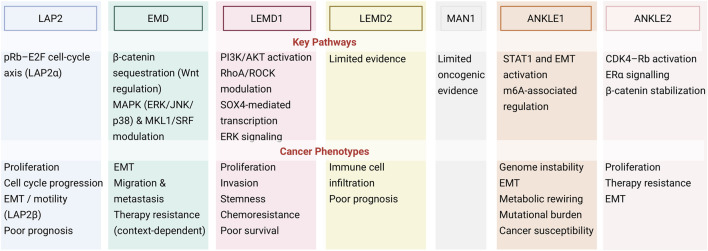
Summary of experimentally supported signaling pathways and cancer-related phenotypes associated with LEM-D proteins. Created with BioRender.com.

Unlike ANKLE1, which functions as an endonuclease, ANKLE2 serves as a scaffold facilitating various protein-protein interactions and is involved in maintaining NE stability. In high-grade serous ovarian carcinoma, ANKLE2 is among a group of 12 genes that engage with VIRMA, a gene that enhances tumor aggressiveness through N6-methylation of adenosine (m6A) (Miranda-Gonçalves et al., 2021). Suppression of ANKLE2 through RNA interference in different human ovarian cancer cell lines (SKOV3, OVCAR, and APOCC) reduced viability and migration, and heightened sensitivity to paclitaxel (Al-Farsi et al., 2022). Similarly, in estrogen receptor-positive (ESR+) human breast cancer cell lines, overexpression of ANKLE2 has been associated with enhanced tumor progression. Mechanistically, ANKLE2 supports the phosphorylation of the tumor suppressor protein Rb and activation the cyclin D-CDK4-Rb; and is associated with tamoxifen resistance (Gao et al., 2018). In ESR + human breast cancer cells, ANKLE2 acts as a scaffold promoting estrogen receptor alpha (ESRα) phosphorylation through aurora-A kinase, thereby activating Erα transcriptional activity. More recently, Tang et al. (2025) reported that ANKLE2 upregulation is associated with acquired resistance to the CDK4/6 inhibitor ribociclib in breast cancer models. ANKLE2 was shown to stabilize β-catenin, increasing its protein stability, nuclear translocation, and transcriptional activity. Silencing ANKLE2 restored ribociclib sensitivity in resistant cells, and pharmacologic inhibition of β-catenin signaling reversed this resistance phenotype. These findings suggest that ANKLE2 may contribute to CDK4/6 inhibitor resistance through modulation of β-catenin signaling (Tang et al., 2025). Overall, ANKLE2 dysregulation has been linked to tumor growth and therapy resistance in high-grade serous ovarian carcinoma and in breast cancer models. Figure 6 below summarizes the signaling pathways and cancer phenotypes driven by LEM-D proteins.

## 4 Latest progress in therapeutic targeting of LEM-D proteins for cancer treatment

At present, there is no FDA-approved agents that directly target LEM-D proteins. Current strategies primarily involve indirect pharmacologic targeting of downstream signaling pathways and gene-silencing techniques.

Preclinical gene-targeting strategies provide proof-of-concept evidence that LEM-D proteins are functionally targetable. 1n TNBC, siRNA-mediated depletion of EMD, ANKLE2, TMPO, and LEMD2 significantly reduced proliferation and induced apoptosis, while exerting minimal effects on non-malignant cells (Rose et al., 2024). Similarly, RNA interference–mediated suppression of ANKLE2 in ovarian cancer reduced migration and enhanced paclitaxel sensitivity (Al-Farsi et al., 2022). In pancreatic cancer, LEMD1 knockdown inhibited proliferation and invasion while suppressing mTORC1 activation and restoring apoptotic signaling (Cao et al., 2022). In TNBC models, LEMD1 depletion reduced tumor growth and enhanced chemotherapy sensitivity through ERK pathway inhibition (Li et al., 2023).

At present, direct small-molecule inhibitors targeting LEM-D proteins are lacking. However, several studies demonstrate that LEM-D–mediated therapeutic resistance can be pharmacologically reversed by targeting downstream pathways. In ER-positive breast cancer, ANKLE2-mediated tamoxifen resistance was reversed using the CDK4/6 inhibitor palbociclib (Gao et al., 2018). More recently, Tang et al. showed that ANKLE2-mediated ribociclib resistance is mediated through β-catenin stabilization, and pharmacologic β-catenin inhibition restored drug sensitivity (Tang et al., 2025). While other pathway dependencies such as PI3K/AKT activation in LEMD1-high tumors or MAPK/β-catenin signaling in emerin-deficient cancers suggest rational combination strategies, these remain prospective and have not yet been clinically validated.

## 5 Concluding remarks and future perspectives

LEM-D proteins play a central role in nuclear architecture, chromatin organization, and oncogenic signaling, and their dysregulation is increasingly associated with tumor progression and therapeutic resistance. Yet, direct pharmacologic inhibition of LEM-D proteins has not yet been realized. Instead, translational efforts have focused on indirect targeting strategies. Insights from laminopathies indicate that NE dysfunction impairs DNA repair capacity (Bolderson et al., 2019; Gonzalo and Kreienkamp, 2015), supporting potential combination strategies with DNA damage response inhibitors. Additionally, nuclear export inhibition has shown anti-cancer activity in selected malignancies (Shao et al., 2011), although such approaches have not been specifically adopted for LEM-D associated tumors. Much of the current evidence linking LEM-D proteins to cancer is drawn from transcript-level datasets and *in-vitro* models, with limited isoform-specific resolution and insufficiently validated antibodies for precise protein-level and spatial analysis. Additionally, subnuclear localization dynamics and mechanobiological behavior in patient-derived tissues remain underexplored, and *in-vivo* functional validation is limited across tumor types. Addressing these gaps will require integration of proteomic tools, high-resolution imaging, mechanobiology approaches, and lineage-specific *in vivo* models to clarify context-dependent roles of LEM-D proteins in cancer.

Future progress will require better validation of LEM-D proteins at the protein level and more carefully designed clinical studies that take LEM-D protein expression into account.
